# Inhibition of Phosphodiesterase-4 (PDE4) activity triggers luminal apoptosis and AKT dephosphorylation in a 3-D colonic-crypt model

**DOI:** 10.1186/1476-4598-11-46

**Published:** 2012-07-25

**Authors:** Toshiyuki Tsunoda, Takeharu Ota, Takahiro Fujimoto, Keiko Doi, Yoko Tanaka, Yasuhiro Yoshida, Masahiro Ogawa, Hiroshi Matsuzaki, Masato Hamabashiri, Darren R Tyson, Masahide Kuroki, Shingo Miyamoto, Senji Shirasawa

**Affiliations:** 1 Department of Cell Biology, Faculty of Medicine, Fukuoka University, Fukuoka, 814-0180, Japan; 2 Central Research Institute for Advanced Molecular Medicine, Fukuoka University, Fukuoka, 814-0180, Japan; 3 Department of Obstetrics and Gynecology, Faculty of Medicine, Fukuoka University, Fukuoka, 814-0180, Japan; 4 Department of Cancer Biology, Vanderbilt University, Nashville, TN, USA

**Keywords:** PDE4B, Three-dimensional culture, Colorectal cancer, Kras, AKT

## Abstract

**Background:**

We previously established a three-dimensional (3-D) colonic crypt model using HKe3 cells which are human colorectal cancer (CRC) HCT116 cells with a disruption in oncogenic *KRAS,* and revealed the crucial roles of oncogenic KRAS both in inhibition of apoptosis and in disruption of cell polarity; however, the molecular mechanism of KRAS-induced these 3-D specific biological changes remains to be elucidated.

**Results:**

Among the genes that were upregulated by oncogenic KRAS in this model, we focused on the *phosphodiesterase 4B* (*PDE4B*) of which expression levels were found to be higher in clinical tumor samples from CRC patients in comparison to those from healthy control in the public datasets of gene expression analysis. *PDE4B2* was specifically overexpressed among other PDE4 isoforms, and re-expression of oncogenic KRAS in HKe3 cells resulted in *PDE4B* overexpression. Furthermore, the inhibition of PDE4 catalytic activity using rolipram reverted the disorganization of HCT116 cells into the normal physiologic state of the epithelial cell polarity by inducing the apical assembly of ZO-1 (a tight junction marker) and E-cadherin (an adherens junction marker) and by increasing the activity of caspase-3 (an apoptosis marker) in luminal cavities. Notably, rolipram reduced the AKT phosphorylation, which is known to be associated with the disruption of luminal cavity formation and CRC development. Similar results were also obtained using *PDE4B2*-shRNAs. In addition, increased expression of *PDE4B* mRNA was found to be correlated with relapsed CRC in a public datasets of gene expression analysis.

**Conclusions:**

These results collectively suggested that PDE4B is upregulated by oncogenic KRAS, and also that the inhibition of PDE4 catalytic activity can induce both epithelial cell polarity and luminal apoptosis in CRC, thus highlighting the utility of our 3-D culture (3 DC) model for the KRAS-induced development of CRC in 3-D microenvironment. Indeed, using this model, we found that PDE4B is a promising candidate for a therapeutic target as well as prognostic molecular marker in CRC. Further elucidation of the signaling network of PDE4B2 in 3 DC would provide a better understanding of CRC *in vivo*.

## Background

Both cell-cell and cell-extracellular matrix interactions are critically involved in developmental programs and provide three-dimensional (3-D) architectures *in vivo*[1], and deregulation of these interactions is frequently observed in cancer [2]. Human cancers are derived from epithelial tissues characterized by specific cellular architectures including epithelial cell-cell junctions, which allow the separation of apical and basolateral membranes. This apical-basal cell polarity is crucial in normal cell functions, and loss of cell polarity is a critical step in tumorigenesis [3].

We recently demonstrated that HKe3 cell line, which is a human colorectal cancer (CRC) HCT116 cell line disrupted at oncogenic *KRAS*[4], in 3-D Matrigel culture (3 DC) manifests an organized structure resembling a colonic crypt [5]. In this model, oncogenic KRAS was found to be involved in the inhibition of luminal apoptosis, impairment of epithelial cell polarity and downregulation of DNA repair genes including *TP53* and *BRCA2* in a 3-D specific manner [5], suggesting that this model could mimic the *in vivo* growth of the colonic epithelium and would be useful for determining the critical genes involved in CRC development through oncogenic KRAS-mediated signals *in vivo*.

We previously identified phosphodiesterase 4B (PDE4B) as one of the differentially expressed between HCT116 cells and HKe3 cells in this model [5]. PDE4 cyclic AMP (cAMP)-specific phosphodiesterase family members are hydrolytic enzymes responsible for the degradation of the second messenger cAMP in many cell types, and the family consists of four genes (PDE4A, PDE4B, PDE4C and PDE4D) encoding multiple isoforms [6-8]. These isoforms can have unique functional roles by their targeting to specific signaling complexes where they underpin the compartmentalization of cAMP signaling [9]. Notably, particular PDE4 isoforms are subject to different regulatory influences, such as phosphorylation [10], ubiquitination [11] and activity changes induced by interacting proteins [12]. For example, the interaction of the disrupted in schizophrenia 1 (DISC1) with PDE4B1 or PDE4B3 and mutations in DISC1 associates with schizophrenia [13].

PDE4 is ubiquitously expressed in inflammatory cells and PDE4 inhibitors have a therapeutic potential for inflammatory diseases, including asthma, chronic obstructive pulmonary diseases, inflammatory bowel disease and psoriasis [14]. A recent study suggests that overexpression of PDE4 enzymes is critical for the MAPK activation by oncogenic-RAS in melanoma cells [15], indicating the novel strategy targeting PDE4 activity in melanoma cells with oncogenic KRAS. However, the precise mechanism of PDE4B in 3-D microenvironment of CRC with oncogenic KRAS has not been addressed so far.

In this study, we used a selective inhibitor of all PDE4 sub-families, rolipram [9,14,16] or *PDE4B2-*shRNAs, and found that rolipram and *PDE4B2*-shRNAs revert the disorganization of CRC into the normal physiologic state of the epithelial cell in 3 DC.

## Results

### Commonly upregulated genes between HCT116 cells in 3 DC and clinical CRC samples

**Figure 1:**
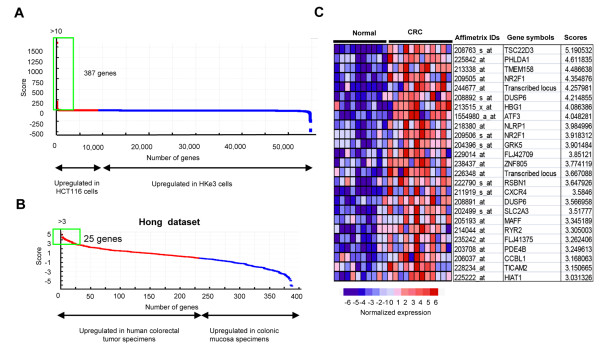
**Commonly upregulated genes between HCT116 cells in 3 DC and clinical CRC samples.(A)** Differentially expressed genes between HCT116 and HKe3 cells grown in 3 DC for 6 days. Dots represent the corresponding score for each gene (red, the genes upregulated in HCT116 cells; blue, the genes upregulated in HKe3 cells). The green square represents the genes with a score of more than 10 (387 genes). **(B)** Differential expressions of the 387 genes detected in (A) between 12 human colorectal tumor specimens and 10 colonic mucosa specimens from the Hong dataset. Dots represent the corresponding score of each gene (red, the genes upregulated in CRC; blue, the genes upregulated in normal colonic mucosa). The green square represents the genes with a score more than three (25 genes). **(C)** The expression pattern of the 25 genes detected in (B) in colorectal tumor specimens and in normal colonic mucosa specimens from the Hong dataset.

To explore the genes regulated by oncogenic KRAS in 3 DC, we analyzed the microarray expression data for HCT116 and HKe3 cells grown in 3 DC on day 6 [5] using the GenePattern software package [17]. We selected the 387 genes with a score of more than 10 as the differentially expressed genes between HCT116 and HKe3 cells grown in 3 DC (Figure 1A, Additional file: 1 Table S1). Then, out of the 387 genes selected, we further selected 25 genes with a score of more than three as the differentially expressed genes between 12 human colorectal specimens and 10 colonic mucosa specimens in a public datasets [18] (Figure 1B, C). Among the 25 genes, we focused on PDE4B, which was suggested to be associated with luminal cavity formation [19].

### PDE4B is upregulated by oncogenic KRAS in 3 DC

**Figure 2:**
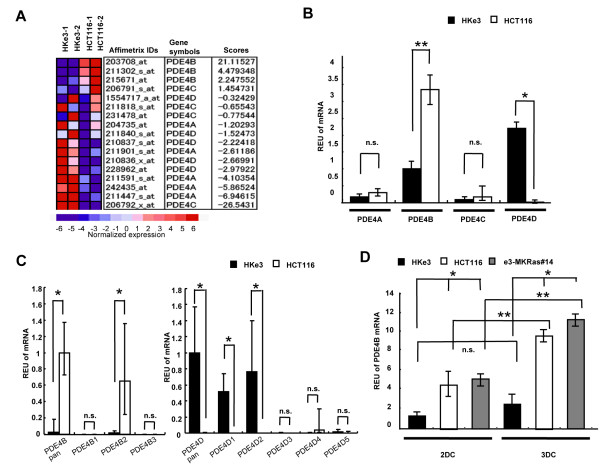
***PDE4B*is upregulated by oncogenic KRAS in 3 DC.(A)** Differential expressions of PDE4 family members between HCT116 cells and HKe3 cells grown in 3 DC. Rows represent Affymetrix IDs, gene symbols and scores. Columns represent normalized expression levels of PDE4 family members. **(B)** mRNA expression levels for PDE4 family members in 3 DC. mRNA expression levels for PDE4 family members in HKe3 (black bar) and HCT116 (white bar) cells grown in 3 DC. *, *P* < 0.05; **, *P* < 0.01; n.s., not significant. **(C)** mRNA expression levels for *PDE4B* and *PDE4D* isoforms in 3 DC. mRNA expression levels for *PDE4B* in HKe3 (black bar) and HCT116 (white bar) cells grown in 3 DC. *, *P* < 0.01; n.s., not significant. **(D)** mRNA expression levels for *PDE4B* in 2 DC or 3 DC. mRNA expression levels for *PDE4B* in HKe3 (black bar), HCT116 (white bar) and e3-MKRas#14 (gray bar) cells grown in 2 DC or 3 DC. *, *P* < 0.05; **, *P* < 0.01; n.s., not significant.

In our microarray expression analyses, the relative expression level of *PDE4B* in 3 DC was increased in HCT116 cells compared with that of HKe3 cells, whereas the relative expression levels of the other PDE4 family members, including *PDE4A*, *PDE4C* and *PDE4D,* were decreased in HCT116 cells in comparison to those in HKe3 cells (Figure 2A).

To confirm these results, quantitative RT-PCR (qRT-PCR) for PDE4 family was performed on HKe3 cells and HCT116 cells in 3 DC. In HKe3 cells, *PDE4B* and *PDE4D* were abundantly expressed than *PDE4A* or *PDE4C*. The expression level of *PDE4B* in HCT116 cells was significantly increased by 3.4-fold in comparison to that in HKe3 cells (**P = 0.0008), whereas the expression level of *PDE4D* in HCT116 cells was significantly decreased by 183-fold in comparison to that in HKe3 cells (Figure 2B; *P = 0.01), indicating that the oncogenic KRAS will regulate PDE4B and PDE4D positively and negatively, respectively.

To elucidate which isoform of PDE4B or PDE4D is exactly expressed, qRT-PCR was performed on HKe3 cells and HCT116 cells in 3 DC. Among PDE4B and PDE4D isoforms, the expression level of *PDE4B2* in HCT116 cells was only increased in comparison to that in HKe3 cells (Figure 2C; *P < 0.01). On the other hand, the expression levels of *PDE4D1* and *PDE4D2* in HCT116 cells were significantly decreased in comparison to these in HKe3 cells (Figure 2C; *P < 0.01), indicating that the oncogenic KRAS will positively regulate PDE4B2, and negatively regulate PDE4D1 and PDE4D2.

To confirm the oncogenic KRAS-mediated upregulation of *PDE4B*, qRT-PCR was performed on HKe3 cells, HCT116 cells and e3-MKRas #14 cells re-expressing oncogenic KRAS. In two-dimensional culture (2 DC), the expression levels of *PDE4B* in HCT116 cells and e3-MKRas#14 cells were increased by 3.6- and 4.0-fold (*P < 0.05), respectively, in comparison to that in the HKe3 cells (Figure 2D). In 3 DC, the expression levels of *PDE4B* in HCT116 cells and e3-MKRas#14 cells were increased by 7.3- and 11.2-fold (*P < 0.05), respectively, in comparison to that in the HKe3 cells (Figure 2D). These results suggest that oncogenic KRAS in two dimensional (2-D) and 3-D cultures upregulates the mRNA expression of *PDE4B.* Furthermore, the expression levels of *PDE4B* in HCT116 and e3-MKRas#14 cells in 3 DC were increased by 2.0- and 2.8-fold (**P < 0.01), respectively, in comparison to those in 2 DC, whereas the expression level of *PDE4B* in the HKe3 cells in 3 DC was not significantly different in comparison to that in 2 DC (Figure 2D). These results together suggest that PDE4B, especially PDE4B2, plays particular roles in the 3-D microenvironment.

### Formation of luminal cavities and tight junctions after treatment with PDE4 inhibitor in HCT116 cells

**Figure 3:**
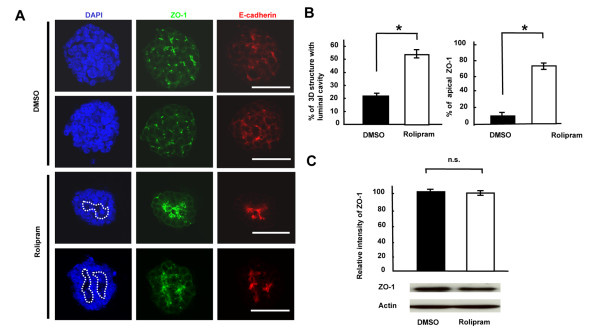
**Formation of luminal cavities and tight junctions by PDE4 inhibitor in HCT116 cells grown in 3 DC.(A)** The signals for ZO-1 and E-cadherin in HCT116 cells treated with DMSO alone or rolipram at day 6 in 3 DC. Nuclear DNA, blue; ZO-1, green; E-cadherin, red. Dots represent luminal cavities. Scale bar = 50 μm. **(B)** The ratios of 3-D structures with luminal cavity (left panel) and apical ZO-1 signal (right panel). White bar represents a relative intensity of the signal for 3 DC HCT116 cells treated with rolipram, normalized by the signal for 3 DC HCT116 cells treated with DMSO alone (black bar). *, *P* < 0.0001. **(C)** Western blotting of ZO-1 for 3 DC HCT116 cells treated with DMSO alone or rolipram at Day 6 (lower panel) and the quantitative analysis of ZO-1 expression level (upper panel). White bar represents a relative intensity of the signal for 3 DC HCT116 cells treated with rolipram normalized by the signal for 3 DC HCT116 cells treated with DMSO alone (black bar). n.s., not significant.

To address the roles of PDE4B in cell polarity, ZO-1 (a tight junction marker) and E-cadherin (an adherens junction marker) were immunostained in HCT116 cells grown in 3 DC treated with rolipram (PDE4 inhibitor) or DMSO alone. The ZO-1 and E-cadherin assembly at the apical surface of acini was more clearly observed in HCT116 cells in 3 DC treated with rolipram in comparison to that in DMSO alone (Figure 3A). These results indicated that rolipram induces the formation of the junctional complexes essential for the maintenance of the physiologic epithelial cell polarity [20] in HCT116 cells grown in 3 DC.

To confirm these results, the quantitative assays were performed. The ratio of 3-D structures with luminal cavities in HCT116 cells treated with rolipram was increased by 2.46-fold in comparison to those treated with DMSO alone (Figure 3B left panel; *P = 0.00009), thus suggesting that rolipram induces the formation of the luminal cavity. The ratio of the 3-D structures with the concentrated signals for ZO-1 in the apical region of the HCT116 cells treated with rolipram increased by 7.67-fold in comparison to those treated with DMSO alone (Figure 3B right panel; *P = 0.00002), thus suggesting that rolipram induces the formation of tight junctions. As the expression levels of ZO-1 were not significantly different between HCT116 cells treated with DMSO alone or rolipram (Figure 3C; *P* = 0.09), ZO-1 was properly translocated to the tight junction at the apical surface of the 3-D structures by reduction of the activity of PDE4B.

### Induction of luminal apoptosis by rolipram in HCT116 cells grown in 3 DC

**Figure 4:**
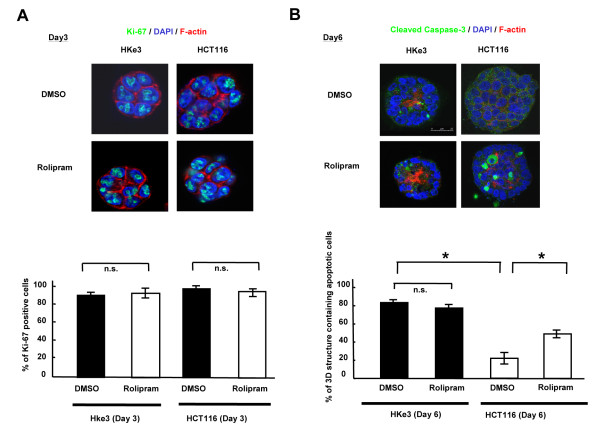
**Induction of luminal apoptosis by rolipram in HCT116 cells grown in 3 DC.(A)** The signals for Ki-67 in HCT116 and HKe3 cells treated with DMSO alone or rolipram at day 3 in 3 DC. Ki-67, green; F-actin, red; nuclear DNA (DAPI), blue. Scale bar = 50 μm (upper panels). The ratio of Ki-67-positive cells in 3-D structures (lower panel). Black bar, 3-D structures treated with DMSO alone; white bar, 3-D structures treated with rolipram. n.s., not significant. **(B)** The signals for cleaved caspase-3 in HCT116 and HKe3 cells treated with DMSO alone or rolipram at day 6 in 3 DC (upper panels). F-actin, red; DAPI, blue; cleaved caspase-3, green. Scale bar = 50 μm. The ratio of the 3-D structures containing apoptotic cells (lower panel). Black bar, 3-D structures treated with DMSO alone; white bar, 3-D structures treated with rolipram. *, *P* < 0.001; n.s., not significant.

To address the roles of PDE4B in the process of colonic crypt organization, we compared the differences in cell proliferation and luminal apoptosis between HKe3 and HCT116 cells grown in 3 DC treated with rolipram or DMSO alone. The proliferation rate detected by Ki-67 staining in 3 DC on day 3 was not different between HKe3 cells treated with DMSO alone and those treated with rolipram (Figure 4A). Similar result was obtained in HCT116 cells treated with DMSO alone and those treated with rolipram (Figure 4A). These results indicated that rolipram does not affect cell proliferation in HKe3 or HCT116 cells grown in 3 DC on day 3.

To address whether rolipram influences luminal apoptosis in 3 DC, we evaluated the apoptotic activity in HKe3 and HCT116 cells grown in 3 DC for 6 days with DMSO alone or rolipram by detecting the signals for cleaved caspase-3 in each 3-D structure using confocal microscopy. Without the treatment of rolipram, the ratio of the 3-D structures containing apoptotic cells in HCT116 cells was decreased by 3.77-fold in comparison to that for HKe3 cells (Figure 4B; *P = 0.00002), which observation was concordant with the previous report [5]. The ratios of the 3-D structures containing apoptotic cells in HKe3 cells treated with DMSO alone or rolipram were not different (Figure 4B; *P = 0.25), suggesting that rolipram does not further affect luminal apoptosis in the cells already having developed luminal cavity. On the other hand, the ratio of the 3-D structures containing apoptotic cells in the HCT116 cells treated with rolipram was found to be increased by 2.21-fold in comparison to those treated with DMSO alone (Figure 4B; *P = 0.0004), suggesting that a physiological apoptosis can be restored by the inhibition of PDE4 catalytic activity.

### Induction of luminal apoptosis by *PDE4B2*-shRNAs in HCT116 cells grown in 3 DC

**Figure 5:**
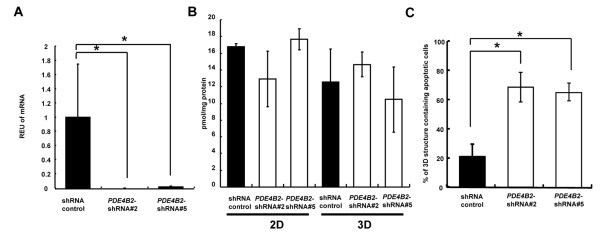
**Induction of luminal apoptosis by*PDE4B2*-shRNAs in HCT116 cells grown in 3 DC.(A)** mRNA expression levels for *PDE4B2* in 3 DC. *, *P* < 0.005. **(B)** The amount of cytoplasmic cAMP in HCT 116 cells grown in 2-D or 3-D culture. **(C)** The ratio of the 3-D structures containing apoptotic cells. *, *P* < 0.005. mRNA expression levels for PDE4B2 in HCT116 cells with control shRNA (black bar) and *PDE4B2*-shRNAs (white bar) grown in 3 DC.

To asses the direct role of PDE4B2 gene, we established stable HCT116 transfectants expressing control shRNA or *PDE4B2*-shRNAs through the use of lentivirus*.* A qRT-PCR analysis showed that the expression level of *PDE4B2* mRNA in the HCT116 cells expressing the *PDE4B2*-shRNA #2 and #5 were significantly decreased compared with those in HCT116 cells transfected with the control shRNA (Figure 5A; *P < 0.005). To examine whether *PDE4B2*-shRNAs affect cytoplasmic cAMP level, we performed the enzyme-linked immunosorbent assay for cAMP in these HCT116 cells transfected with control shRNA or PDE4B2-shRNAs in 2-D or 3-D culture. The expected cAMP levels were detected in all cell lines, however, the reduced expression of *PDE4B2* did not affect cytoplasmic cAMP levels as reported before [21] in 2-D or 3-D culture (Figure 5B). To address whether *PDE4B2*-shRNAs influence luminal apoptosis in 3 DC, we evaluated the apoptotic activity in these cells grown in 3 DC for 6 days by detecting the signals for cleaved caspase-3 using confocal microscopy. In HCT116 cells with control shRNA, the ratio of the 3-D structures containing apoptotic cells was decreased by 3.30- and 3.13-fold in comparison to that for HCT116 cells with *PDE4B2*-shRNA #2 and #5, respectively (Figure 5C; *P < 0.005). This observation was similar with the result obtained in Figure 3B, suggesting that a physiological apoptosis can be restored by the direct inhibition of expression level of *PDE4B2* mRNA.

### 3-D-specific reduction in AKT phosphorylation by rolipram or *PDE4B2*-shRNAs in HCT116 cells

**Figure 6:**
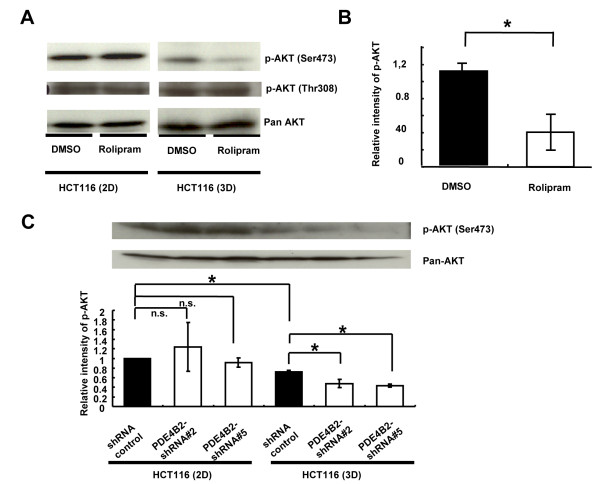
**Reduction of AKT phosphorylation by rolipram in HCT116 cells grown in 3 DC.(A)** Western blotting of AKT phosphorylation (p-AKT) at Ser473 and Thr308, and pan-AKT for HCT116 cells treated with DMSO alone or rolipram at day 6 in 2-D or 3-D culture. **(B)** The quantitative analyses of the level of p-AKT (Ser473) in HCT116 cells treated with DMSO alone or rolipram at day 6 in 3 DC. White bar represents the relative intensity of the signal for HCT116 cells treated with rolipram, normalized by the signal for HCT116 cells treated with DMSO alone (black bar). *, *P* < 0.01. **(C)** Upper panel shows western blotting of p-AKT (Ser473) and pan-AKT for HCT116 cells stably transfected with control shRNA or *PDE4B2*-shRNAs at day 6 in 2-D or 3-D culture. Lower panel shows the quantitative analyses of the level of p-AKT in HCT116 cells stably transfected with control shRNA or *PDE4B2*-shRNAs at day 6 in 3 DC. White bar represents the relative intensity of the signal for HCT116 cells stably transfected with *PDE4B2*-shRNAs, normalized by the signal for HCT116 cells stably transfected with control shRNA (black bar). *P < 0.05.

AKT is reported to play a major role in regulating acinar structure [22], and PDE4B is suggested to be a selective modulator for AKT phosphorylation at Ser473 [23]. Therefore, we examined the effect of rolipram on phosphorylation of AKT at S473, which is associated with enhanced AKT activity, in CRC cells in 2 DC and 3 DC. While the level of the AKT phosphorylation in HCT116 cells in 2 DC was not affected by rolipram, its phosphorylation was decreased by 2.70-fold in the HCT116 cells grown in 3 DC in response to rolipram treatment (Figure 6A and 6B; *P = 0.003). Additionally, we also evaluated the phosphorylation of AKT at Thr308, however, any significant difference was not observed (Figure 5A). Similarly, the level of the AKT phosphorylation at Ser473 in HCT116 cells in 2 DC was not affected by *PDE4B2* –shRNAs. On the other hand, the levels of AKT phosphorylation at Ser473 were decreased by 1.51- and 1.67-fold in the HCT116 cells with *PDE4B2*-shRNA #2 and #5 grown in 3 DC, respectively (Figure 6C; *P < 0.05). These results suggested that the oncogenic KRAS will disrupt the acinar structure, in part, through the regulation of AKT phosphorylation by increasing the activity of PDE4B in the 3-D microenvironment.

### Correlation between increased *PDE4B* expression and disease relapse in CRC patients

Recent studies indicated that 3 DC mimics the early step of metastasis process [24] and the differentially expressed genes between organized and unorganized multicellular structures grown in 3 DC share similarities with the differentially expressed genes between good- and poor-prognosis tumors [25-27]. To examine the correlation between PDE4B expression and the prognosis of clinical colorectal tumors, we analyzed 18 genes out of 25 KRAS-upregulated genes (Figure 1B,C) in a public datasets of the microarray-based gene expression analyses of human colorectal tumor specimens from 6 CRC patients in the relapsed group and those from 10 patients in non-relapsed group in a public datasets [28], revealing that expression of *PDE4B* mRNA was significantly upregulated in the relapsed group compared with the non-relapsed group (Figure 6), thus suggesting the critical involvement of PDE4B in tumor progression and poor prognosis.

## Discussion

In polarized cells, the inactivation of AKT is thought to be important for the lumen formation with apoptosis in 3 DC [22,29,30]. The findings of AKT dephosphorylation by PDE4 inhibitor, rolipram or *PDE4B2*-shRNAs in HCT116 cells in 3 DC, but not in HCT116 cells in 2 DC (Figure 4 and 5), suggesting the 3-D specific action of PDE4B2 for cancer cells with oncogenic KRAS.

**Figure 7:**
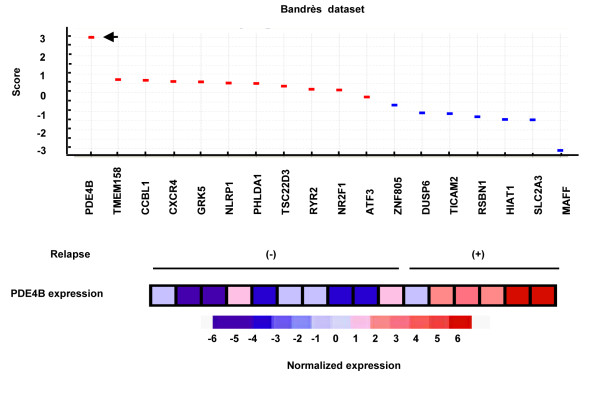
**Correlation between increased PDE4B expression and disease relapse in CRC patients.** Differential expressions of 18 genes between colorectal tumor specimens from six CRC patients in the relapsed group and those from 10 patients in non-relapsed group in Bandrès dataset. Dots in upper panel represent the score (red, upregulated genes in relapsed group; blue, upregulated genes in non-relapsed group). Arrowhead represents PDE4B. Columns in lower panel represent normalized expression levels of *PDE4B* in 16 Dukes' B colorectal tumor specimens.

In clinical samples, increased expression level of *PDE4B* mRNA was correlated with disease relapse in CRC patients (Figure 7). Furthermore, among 25 KRAS-upregulated genes (Figure 1B,C), the predictive power of PDE4B expression for poor prognosis is stronger than that of CXCR4 which is reported to be a prognostic factor for poor disease outcome [31,32]. PDE4B is also reported to be predictive of the resistance to EGFR tyrosine kinase inhibitors in human lung tumors with KRAS mutation [33]. These reports indicate that PDE4B is a promising candidate for a prognostic marker in CRC.

Several studies have shown the effectiveness of PDE4 inhibitors. A recent study shows that resveratrol binds with PDE4 and ameliorates aging-related metabolic phenotypes [34], indicating the positive health benefits through the use of PDE4 inhibitors. In cancer, several studies shows the anti-tumor effects of rolipram in combination with forskolin were observed in different types of cancer, including hematological [23,35,36], brain [37,38] and skin cancers [15]. In CRC, Murata et al., reported that the constitutive activation of PDE4 was detected in a colon cancer cell line DLD-1, and rolipram suppressed cellular motility [39]. McEwan et al., reported that the 10 μM (the same dose used in our study) of rolipram in combination with an adenylate cyclase activator (forskolin) induced apoptosis in colon cancer cell line KM12C [40]. These reports suggested that the apoptotic reaction was induced through the increased stability of cAMP by PDE4 inhibitor or the increased activity of cAMP by forskolin [40]. Notably, our study demonstrated that luminal apoptosis was effectively induced by rolipram alone (Figure 4) or *PDE4B2*-shRNAs (Figure 5) without affecting cytoplasmic cAMP level (Figure 5B) in HCT116 cells in 3 DC, suggesting that the effect of PDE4 inhibitor is enhanced by 3-D microenvironment and independent of cAMP level. Indeed, ablation of PDE4B, but not PDE4A or PDE4D, causes a major decrease in TNF production and global change in cAMP could not be detected in PDE4B^−/−^ macrophage, together, suggesting PDE4B2 may have specific role among PDE4B isoforms. For example, PDE4B2 is highly expressed in immune cell and associated with diffuse large B cell lymphoma [35]. In melanoma, the increased PDE4B2 expression was observed compared with that in melanocyte and PDE4B2 expression is necessary to transform melanocyte with oncogenic NRAS [15], implicating the effective strategy for cancer treatment by targeting the PDE4B2.

## Conclusions

This is a first report about the correlation between the inhibition of PDE4 catalytic activity and luminal cavity formation in CRC cells with oncogenic KRAS grown in 3 DC. Oncogenic KRAS upregulates the *PDE4B* expression and the inhibition of PDE4 catalytic activity by rolipram or the *PDE4B2*-shRNAs triggers the induction of polarity, apoptosis and AKT dephosphorylation in HCT116 cells grown in 3 DC, thus suggesting the increased PDE4B2 activity will be crucial in the inhibition of luminal apoptosis in colorectal epithelium in 3-D microenvironment. Further elucidation of the precise molecular mechanisms of function of PDE4B would provide a better understanding of the development and progression of CRC.

## Methods

### Antibodies and reagents

The anti-Ki-67 (SP6) was obtained from Thermo Scientific (Rockford, IL). The anti-ZO-1 (1/ZO-1) and anti-E-cadherin (36/E-cadherin) were from BD Biosciences (San Jose, CA). Anti-cleaved caspase-3 (5A1), anti-phosphorylated AKT (p-AKT; D9E) and the anti-AKT (pan; C67E7) were from Cell Signaling Technology (Beverly, MA). The anti-actin (A2066), the PDE4 inhibitor, rolipram and 4′,6-diamidino-2-phenylindole (DAPI) were from Sigma-Aldrich (St Louis, MO).

## Cell culture

Human CRC HCT116 cells were obtained from the American Type Culture Collection (Frederick, MD). Two-dimensional (2-D) culture (2 DC) of HCT116 cells, HKe3 cells and e3-MKRas#14 cells (HKe3-derived stable transfectants expressing oncogenic KRAS) was done as described previously [4,5,41]. The 3 DC was performed using Matrigel, a reconstituted basement membrane (Growth Factor Reduced Matrigel; BD bioscience), as described previously [5,42]. All cell lines used were confirmed to be *Mycoplasma*-free, as determined using the MycoAlert system (Lonza, Verviers, Belgium). Cell morphology was regularly checked to ensure the absence of cross-contamination of cell lines.

## Microarray expression analysis

Total RNA from cells in 3 DC was extracted as described previously [5,43]. Human Genome U133 Plus 2.0 Array 6800 GeneChips (Affymetrix, Santa Clara, CA) containing 54,675 oligonucleotides were processed with the use of robust multi-chip averaging [44]. The differentially expressed genes between two classes were ranked according to signal-to-noise metric with the GenePattern software package [17,45]. The statistical significance of the differentially expressed genes was determined by the comparative marker selection module in Gene-Pattern [17].

## Dataset source

The Hong dataset, consisting of the microarray profiles of human colorectal tumor specimens from 12 CRC patients and colonic mucosa specimens from 10 healthy control subjects [18], was obtained from the Gene Expression Omnibus (GEO; Series GSE4107). The Bandrès dataset, consisting of human Dukes' B colorectal tumors from 16 CRC patients with associated clinical data [28], was also obtained from the GEO (Series GSE2630). GEO series records were imported by the import module in the GenePattern software package [17]. The platform used for Hong dataset was Human Genome U133 Plus 2.0 Array 6800 GeneChips (Affymetrix), which was identical to our previous study [5]. As the platform used for Bandrès dataset was Human 19 K Oligo array slides (Center for Applied Genomics, University of Medicine of New Jersey), 25 probe set IDs in Human Genome U133 Plus 2.0 Array 6800 GeneChips (Affymetrix) were converted to gene symbols and 18 genes were identified out of the 25 probe set IDs.

## Quantitative RT-PCR

Quantitative RT-PCR was performed using ABI PRISM 7900HT (Applied Biosystems, Carlsbad, CA) as described previously [5,43]. The PCR primer sequences used for PDE4 isoforms and the accession numbers for the genes are listed in Additional file: 2 Table S2. The PCR primers used for PDE4A (pan), PDE4C (pan) and PDE4D (pan) were designed to amplify a 3’ fragment found in all PDE4 sub-families as reported before [7]. The PCR primers used for PDE4B (pan) were designed to amplify a fragment characteristic of human PDE4B1 and PDE4B2 as reported before [7]. The PCR primers used for PDE4B and PDE4D isoforms were designed as described previously [46]. The relative expression unit (REU) values were determined by the setting REU of HKe3 in 2 DC as 1.0.

## Immunofluorescence labeling and confocal microscopy

The immunofluorescence experiments were performed as described previously [5,47]. For the examination of 3-D structures, TCS-SP5 Laser Scanning confocal microscopy (Leica, Wetzlar, Germany) was used.

## Quantification of apical ZO-1 signals and luminal cavities in 3-D structures

The cells were stained with an anti-ZO-1, anti-E-cadherin antibody and DAPI on day 6, and the ratio of 3-D structures with ZO-1 signals and the luminal cavities in the apical region were counted as described previously [5]. A total of 60 of the 3-D structures from three different wells were counted.

## Quantification of proliferative cells grown in 3 DC

The cells were stained with the anti-Ki-67 antibody, DAPI and phalloidin on day 3. The ratio of Ki-67-positive cells in the total cells contacting matrigel in the cross-section of 3-D structures at maximum diameter was calculated as described previously [5]. The number of 3-D structures evaluated for each condition was 20.

## Quantification of apoptosis in 3-D structures

The cells were stained by anti-cleaved caspase-3 antibody, DAPI and phalloidin on day 6. The cleaved caspase-3-positive cells in 3-D structures were counted in the serial cross-sections of the 3-D structure ranged from 60 to 130 μm in the maximum diameter. The 3-D structures containing more than two positive cells with luminal cavity or actin assembly at the apical surface of acini were defined as the 3-D structure containing apoptotic cells. A total of 60 of the 3-D structures from three different wells were counted. The 3-D structures of HCT116 and HKe3 cells were analyzed in three independent experiments, and the average ratio of the 3-D structures containing apoptotic cells was calculated as described previously [5].

## Western blotting analysis

The western blotting analyses were performed as described previously [5]. The actin intensity was used as a control in the western blot analyses for ZO-1, and the relative intensity of the signal (ZO-1/Actin) was normalized to the signal-intensity in HCT116 cells treated with DMSO alone (as 100%). Pan-AKT intensity was used as a control in the western blotting analyses for p-AKT, and the relative intensity of the signal (p-AKT/pan-AKT) was normalized to the signal intensity in HCT116 cells treated with DMSO alone as 100%.

## Generation of lentivirus vectors expressing *PDE4B2*-shRNAs

The short hairpin interfering RNA (shRNA) targeting GFP was used as a control. For *PDE4B2* knockdown, shRNAs were designed based on the sequence information from PDE4B2-siRNAs used in the study for diffuse large B-cell lymphoma [35]. The shRNA duplexes used were: *PDE4B2-*shRNA #2 top, 5’-CAC CGC CTA AAC AAT ACA AGC ATT TCA AGA GAA TGC TTG TAT TGT TTA GGC-3’ and *PDE4B2-*shRNA #2 bottom, 5’-AAA AGC CTA AAC AAT ACA AGC ATT CTC TTG AAA TGC TTG TAT TGT TTA GGC-3’; *PDE4B2-*shRNA #5 top, 5’-CAC CGC ATC TCA CGC TTT GGA GTT TCA AGA GAA CTC CAA AGC GTG AGA TGC-3’ and *PDE4B2-*shRNA #5 bottom, 5’- AAA AGC ATC TCA CGC TTT GGA GTT CTC TTG AAA CTC CAA AGC GTG AGA TGC-3’. The shRNA expression vectors were constructed as described previously [48]. In brief, The human U6 promoter (Gene bank accession #M14486 gene sequence 65–329) was inserted into *ClaI* and *SalI* sites of the pLenti6/V5-Dest (Invitrogen, Carlsbad, CA, USA), and then U6-term was inserted into the *Sal*I and *Mlu*I sites to form pLenti6-U6 + term. The resulting pLenti6-U6 + term was then cleaved with *BsmB*I to form a cloning site for double-stranded synthetic oligonucleotide DNA.

## shRNA transfection

The shRNA expression vectors were transfected into 293FT cells to produce packaged lentivirus. The lentivirus particles were packaged using the ViraPower Lentiviral Expression System (Invitrogen). The HCT116 cells were then infected with lentivirus *PDE4B2-*shRNAs to obtain stably transfected clones. Blasticidin (Invitrogen) was added to eliminate the cells not expressing *PDE4B2-*shRNAs.

## cAMP analysis

Cytoplasmic protein extraction was performed using NE-PER Nuclear and Cytoplasmic Extraction Reagent (Thermo Scientific) according to the manufacturers instructions. cAMP levels of cytoplasmic extract from 2-D or 3D culture were measured using the direct cAMP ELISA kit (Enzo Life Sciences, Farmingdale, NY, USA) according to the manufacturer’s instructions.

## Statistical analysis

The data are presented as the means ± standard deviation. The statistical analyses were performed using unpaired two-tailed Student’s *t-*test. Differences at *P* < 0.05 were considered to be statistically significant.

## Abbreviations

KRAS, v-Ki-ras 2 Kirsten rat sarcoma viral oncogene homolog; AKT, Akt murine thymoma viral oncogene homolog; TP53, Tumor protein 53; BRCA2, Breast cancer gene 2; MAPK, Mitogen-activated protein kinase; ZO-1, Zonula occludens-1; EGFR, Epidermal growth factor receptor.

## Competing interests

The authors declare that they have no competing interests.

## Authors’ contributions

TT performed the statistical analysis, coordinated the experiments, and drafted the manuscript. TO carried out the cell and molecular biology experiments and drafted the manuscript. TF, YT, YY, MO and HM performed the cellular assays, molecular studies, western blots and RT-PCR. KD carried out the microarray gene expression analysis and bioinformatics. DRT contributed interpretation and analyzed data. MK and SM participated in study design. SS contributed to data evaluation, interpretation and drafted the manuscript. All authors have read and approved the final manuscript.

## Supplementary Material

Additional file 1 **Additional Table 1.** Upregulated genes by oncogenic KRAS in 3 DC (HCT116/HKe3 in 3 DC; Score > 10.00; 387 genes). Click here for file

Additional file 2 **Additional Table 2.** List of the forward and reverse primers used to amplify PDE4 variants and β-actin. Click here for file
